# Determinants of suicidal behavior among elders in Northwest Ethiopia: implications for prevention

**DOI:** 10.3389/fpsyt.2025.1538877

**Published:** 2025-02-24

**Authors:** Seid Siraj, Habte Belete, Michael Beka, Maregu Shegaw, Asmare Belete, Zelalem Birhan

**Affiliations:** ^1^ Psychiatry Unit, Akasta General Hospital, Akasta, Ethiopia; ^2^ Department of Psychiatry, College of Medicine and Health Science, Bahirdar University, Bahirdar, Ethiopia; ^3^ Department of Psychiatry, College of Medicine and Health Science, Wollo University, Dessie, Ethiopia

**Keywords:** elders, Ethiopia, suicidal attempts, suicidal behavior, suicidal ideation

## Abstract

**Background:**

Worldwide suicide rates increases with age. Globally, suicidal behavior is a leading cause of injury and death. In many countries, older adult suicidal behavior is highly lethal because old people are unwilling to talk about their emotional problems and are less likely to report depression and suicidal thoughts. Exploring the phenomenon of suicide in the elderly in Ethiopia can provide a dependable source of reflection and add to the global aging, and suicide prevention conversation, generally in the low-income countries and middle-income countries (LMICs). This study aimed to assess the prevalence of suicidal behavior and its associated factors among elders in Bahir Dar city, Northwest Ethiopia.

**Method:**

A community-based cross-sectional study and multistage sampling technique were conducted among elders in Bahir Dar city. A systematic random sampling procedure was used to choose 626 elderly people over 65 years old in total who had lived in Bahir Dar city. Utilizing the revised Suicidal Behavior Questionnaire (SBQ-R), suicidal behavior was assessed. We quantify the related components using multivariable logistic regressions. The factors’ associations were delineated with odds ratios, 95% confidence intervals, and p-values that were deemed statistically significant at less than 0.05.

**Result:**

Overall, 12.8% (95% CI: 10.2, 15.3) of the population engaged in suicidal behaviors. The lifetime prevalence of suicidal ideation, plan, and attempts were 13.9%, 8.15%, and 1%, respectively. The prevalence of suicidal ideation in the past 12 months was 10.86%. The odds of being single (AOR: 2.19, 95% CI: 1.18, 4.06), having no social networks (AOR: 2.25, 95% CI: 1.01, 5.01), being depressed (AOR: 4.01, 95% CI: 1.97, 8.17), having a chronic illness (AOR: 3.03, 95% CI: 1.69, 5.44), and geriatric mistreatment (AOR: 7.81, 95% CI: 4.06, 15.05) were the independent predictors of suicidal behavior.

**Conclusion:**

The extent of suicidal behavior was found to be high in this study. The associated factors of suicide behavior include being unmarried, having a poor social network, having depression, chronic illness, and geriatric mistreatments. Therefore, clinicians should do routine mental health examinations for older persons, focusing on those who have a history of elder abuse or chronic illnesses, as these are major risk factors for suicide behavior. To detect and treat suicidal thoughts in elder populations, healthcare professionals should get culturally appropriate training. For legislators: create policies that address elder abuse by instituting community reporting mechanisms and legal protections for elder citizens, and give top priority to developing national healthcare initiatives that include elder-specific mental health and suicide prevention programs.

## Introduction

Suicidal behavior includes a variety of self-harming thoughts and behaviors, such as suicidal ideation (thinking about, considering, or planning suicide), suicide attempts (doing something that could harm oneself with the intention of dying), and completed suicide (death brought on by self-directed harmful behavior with the intention of dying) (1). Three categories exist for suicidal behavior: suicidal thought, suicidal plan or intent, and suicidal attempts. The belief that one is acting as their own death’s agent is known as suicidal ideation; the degree of suicidal purpose and the complexity of one’s preparations determine how serious an ideation is. Suicidal intent is the subjective hope that one will die from a self-destructive act. Self-injurious activity with a nonfatal consequence and either overt or covert indications that the person meant to die is known as a suicide attempt (2).

According to a 2017 World Health Organization (WHO) estimate, 800,000 people die by suicide each year (3). Suicide is a very big problem almost in the whole world, and around 703,000 deaths reported annually due to suicide, and many more individuals engaging in non-fatal suicidal behaviors and among the elderly population, suicidal behavior is particularly alarming due to its high lethality and often undetected nature (4). Approximately 78% of all suicides that are completed worldwide take place in low- and middle-income nations (5). According to US polls conducted in 2017, there were over 42,000 suicide attempts annually across all age groups (6). Later-life suicide is a worldwide public health concern, with most nations having the greatest suicide rate among people 65 and older (7).

The worldwide incidence of suicide increases with age, with the rate of suicide in those aged over 75 years reaching up to twice or three times the rate in those under 25 years in most countries (8). Older adults in most countries constituting those 85-90 years age group have the highest prevalence of suicidal behavior and suicidal rate (9). Therefore, relative to the younger age groups, older people have a greater chance of dying by suicide in developing countries (10). The worldwide suicide rate for both men and women expands inexorably with age, reaching its highest peaks in the 85 and older age group (11).

Loneliness, poor family connections, and inflammation, neurodegeneration, and hypothalamic-pituitary-adrenal (HPA) axis dysregulation are contributors to late-life depression and suicide risk. Additionally, chronic diseases such as diabetes, cardiovascular disease, and chronic pain conditions bring additional harm to the mind, thus worsening the suicides in the elderly (12).

The occurrence of suicide amongst the elderly was high, with the change ranging from 2.2% to 21.5% (13). WHO in 2017, showed that suicide in elders occurs almost equally in high- and low-income countries (14). Globally, women are about three times as likely as men to attempt suicide, yet men are significantly more likely to complete suicide due to the use of more lethal methods (15). However, it remains unclear whether this pattern is consistent among the elderly population or in low- and middle-income countries (LMICs) like Ethiopia. However, old age is markedly characterized by diverse losses for many elderly people: physiological, functional, social, cognitive, financial, and environmental isolation, a subjective sense of loneliness; anxiety; depression, and frequently loss of motivation to continue living often arise from such losses (16).

Suicidal ideation significantly increases the risk of suicide attempts and completions (17). In the first year after the beginning of ideation 60% of changes from ideation to preparation and attempt occur (18). The most frequent reasons for suicidal ideation are diseases or disorders, the second most frequent was ‘loneliness’ (17.2%), and the third was financial problems (11.9%) (19). Suicide attempts among the elderly are more likely to be lethal due to several factors, notably the presence of physical health factors, alcohol abuse, stressful life events, social isolation, and psychiatric disorders (especially depressive disorders) (20), whereas, socio-economic status, marital status, physical health, mental health, influence of major events, religious belief and social interaction, poverty, lack of social support, and untreated mental health conditions are mainly the factors responsible for suicidal ideation for the elderly population (21, 22).

However, suicides are frequently underreported or incorrectly classified as accidents or natural deaths due to religious and societal standards; this pattern has also been seen in other LMICs. Suicide prevention initiatives are made more difficult by these cultural barriers, which impede reliable data collecting and public health measures (23). Specifically, Ethiopia’s unique societal, religious, and economic characteristics make it a unique place to investigate suicide behavior in older populations. Suicide is highly stigmatized since it is frequently seen as immoral by dominant cultural and religious ideas. In addition to influencing reporting, this stigma also affects how suicidal conduct is experienced and interpreted. Furthermore, there is an urgent need for context-specific data to guide policy and initiatives due to the nation’s rapidly aging population and the dearth of geriatric and mental health care. Thus, the goal of this study was to evaluate the prevalence of suicide behavior among older people residing in Bahir Dar city, Northwest Ethiopia, as well as the characteristics that are linked to it.

## Methods

### Study area and period

The research was carried out in the Northwest Ethiopian city of Bahir Dar between March 10 to April 18, 2021. The capital of the Amhara regional state, Bahir Dar, is situated 565 kilometers northwest of Ethiopia’s capital, Addis Ababa. According to a 2019 city administration report, from the total population, 15,620 are 65 years and older.

### Study design

A community-based cross-sectional study was conducted.

### Population

The source population consisted of all the elderly people residing in Bahir Dar city, while the study population consisted of the elderly people who were randomly selected from households in the study area during the study period.

### Eligibility criteria

The study included all elderly people over 65 who had lived in Bahir Dar city permanently for more than six months; those who were unable of communicating or who were with severe illness were excluded.

### Sampling size determination

Since no research has been done in Ethiopia, the sample size in this study was determined using a single population proportion formula based on the expected prevalence of suicidal behaviors, which was set at 50%. In order to guarantee sufficient power to identify meaningful correlations between the three main variables (depression, elder abuse, and suicidal conduct), the sample size was determined by a power analysis. A targeted power of 80% and a significance level of α = 0.05 were employed. In order to identify medium-to-large effect sizes (d = 0.5), we therefore calculated that a sample size of 424 participants would be adequate. This estimate was exceeded by the final sample size, which included 636 people in total by applying designing effect, guaranteeing that the study had the power to identify meaningful relationships.

### Sampling procedure

**Figure 1:**
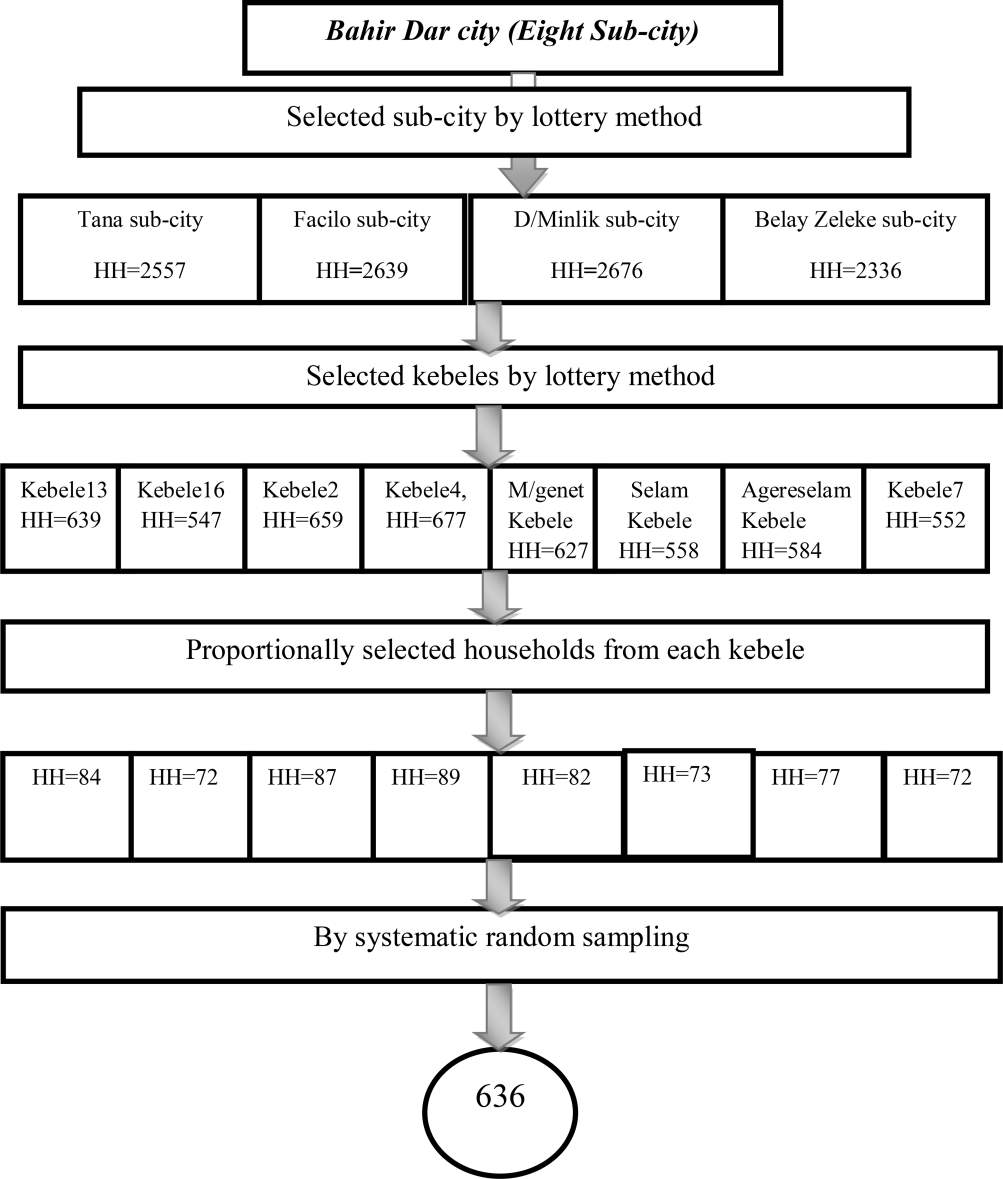
Schematic presentation of sampling technique on the prevalence and associated factors of suicidal behaviors among elders in Bahir Dar city, Northwest Ethiopia, 2021

A method of multi-stage systematic random sampling was employed. Four subcities were chosen at random using lottery techniques from a total of six subcities in the first stage. The sample size was dispersed to the chosen kebele (the smallest administrative unit in Ethiopia, similar to a neighborhood or a community) proportionate to the household size after eight kebeles from a chosen sub-city were chosen by lottery. After determining an initial beginning household through the lottery method, households in the chosen kebele were chosen by systematic random sampling approaches (**Figure 1**).

Eligible participants in the selected household were further selected and interviewed. In cases where there was more than one eligible participant in the household, the lottery method was used to include only one. The interviewer visited the household three times at different times in case the eligible participant was not found at the designated time. If the interviewer was unsuccessful in finding the participant, the household was marked as a non-response. If the selected household did not contain any eligible elders, the next household was chosen.

### Data collection tools and procedures

Four BSc data collectors and one MSc supervisor were chosen from the field of psychiatry, and the primary investigator offered training on data collection methods and instruments. The data was collected by interview-administered questionnaires, and to ensure consistency and understandability, a third party translated a questionnaire from English to Amharic and back to English using language experts. To ensure questionnaire clarity, 32 participants in Adet town outside the study area were given a pretest one week before the actual data collection began. It was ensured that all required data were correctly collected by regular supervision by the primary investigator and the supervisors. Prior to processing and computer entry from paper, the gathered data was thoroughly examined and cleansed.

A structured interviewer-administered questionnaire was used, which has 9 sub-sections: Suicidal behavior was assessed by using the Suicidal Behavior Questionnaire-Revised (SBQ-R), which is broadly used in Ethiopia to screen suicidal behavior (24, 25). It has a sensitivity of 93% and a specificity of 95% with a score of 3-18 and a cutoff point of ≥7 for the non-suicidal or non-clinical sample (26). In this study, the internal consistency was checked and found to have a Cronbach’s α = 0.76. Depression was measured using the Geriatric Depression Scale (GDS) Short Form Scale, which has a standard 15-item cutoff point score of 0-4, which is normal, and a score ≥5 indicates depression. The sensitivity was 81.3%, and the specificity was 78.4% (27).

Chronic diseases were measured by separate ratings of the presence or absence of chronic diseases obtained by asking respondents whether a doctor had ever told them (28). Functional disability was assessed by the Katz scale, which is used to measure the individual’s ability to carry out everyday activities such as bathing, dressing, toileting, transfer, continence, and feeding. Cronbach’s alpha ranged from 0.80 to 0.92 (29).

Nutritional status was measured by using the Mini Nutritional Assessment Short Form (MNA-SF). This tool has 0.85, sensitivity, and 0.87, specificity, with a cutoff point ≤ (11). For body mass index cutoff point ≤ 11, 0.85. Using calf circumference instead of body mass, cutoff points are ≤ 11, and 0.84 (30). It also validated in Ethiopia and had the overall accuracy of the full MNA of 91%. The sensitivity and specificity of the full MNA tool using an established cut-off point were 87.9% and 89.6%, respectively (31).

Quality of life was measured using the 26 items of WHOQOL-BRFE, which is a cross-culturally validated instrument to measure the quality of life, particularly useful when addressing the impact of physical and psychological well-being, but also on several domains beyond health, and had good sensitivity and specificity to assess the quality of life of people in health care settings and community settings (32). There is an Ethiopian validated version of the WHO Quality of Life Human Immune Virus Ethiopia version (WHOQOL-HIV-BREF-Eth) with good psychometric properties (33). The Cronbach alpha was 0.82. QOL scores range between 0 and 100. Scores are scaled in a positive direction (i.e., higher scores correspond to a better health-related quality of life and vice versa).

Social network was measured by LSNS-6, which is a validated instrument designed to gauge social isolation in older adults by measuring the number and frequency of social contacts with friends and family members and the perceived social support received from these sources. Cronbach’s alpha coefficients for family and friend subscales were 0.84 and 0.90, respectively (34). Perceived loneliness was assessed by using the DeJong Gierveld Loneliness Scale, which has a 6-item scale. Three statements are made about ‘emotional loneliness’ and three about social loneliness. The DJGLS showed good internal consistency (Cronbach’s alpha 0.71) and high test-retest reliability (r = 0.93) (35) and the overall loneliness score from 0–6, with higher scores indicating a higher experience of loneliness. Participants were considered to be lonely (score ≥ 2) (36).

Life Events Stressors assessed using yes/no questions about the occurrence of particular stressful life events in the preceding three years. All respondents were asked if they have experienced stressful life events (37). Elder mistreatment can be defined as a single or repeated act or lack of appropriate action occurring within any relationship where there is an expectation of trust, which causes harm or distress to an older person. It can take various forms, such as physical, psychological, sexual, and financial, and it can also be the result of intentional or unintentional neglect (38). And it was assessed by the Geriatric Mistreatment Scale, which was developed in 2013 by Geraldo-Rodriguez and Rosas-Carrasco to assess elder mistreatment, and the Cronbach’s alpha was 0.80 (39). It has 22 items designed to assess five different categories of elder abuse: (a) physical abuse, (b) psychological or emotional abuse, (c) neglect, (d) financial or material abuse, and (e) sexual abuse. The answer to each item is either ‘yes’ or ‘no’, and ‘yes’ for a question equals one point (0=No=No abuse, 1=Yes=Abuse). Each question aims to identify whether there was any mistreatment in the last 12 months, and a ‘yes’ answer to at least one question means that the individual was abused (38). Substance use was assessed by yes/no questions for ever use and current use.

### Data processing and analysis

After the data was coded and checked to be complete, it was imported into Epi-data version 4.6 and exported to SPSS version 25. Adjusted odds ratios and 95% confidence intervals were used to evaluate and show the strength of the relationship between the dependent and independent variables. Data were presented using frequency tables. The final set of confounders was chosen using statistical criteria that took into account both theoretical knowledge and statistical significance. The justification for each confounder’s inclusion is now given, with a focus on how they might affect the association between suicidal conduct and the independent variables (elder abuse, depression). To evaluate the relationship between potential confounders and suicidal behavior, we conducted a number of bivariate studies. For the multivariate logistic regression model, variables that had a significant correlation (p < 0.05) with suicidal behavior were taken into consideration. We performed a multicollinearity check to further improve the model by making sure the included confounders did not show strong correlation and the Hosmer and Lemeshow Test for model fitness, and the result was 0.75, showing that the model fit the data well.

## Results

### Socio-demographic characteristics of the respondents

There were 626 participants in all, yielding a 98.4% response rate. Participants’ median age was 69 (IQR = 5), and 360 (57.51%) of the responses were female. The majority of the respondents were Orthodox in religion (454 (72.52%), married (349 (55.75%), had no formal education (247 (39.56%), were homemakers 203(32.43%), had income above the poverty line 526(84.03%), and 538 (85.94%) were living with their families (**Table 1**).

**Table 1:** Socio-demographic characteristics of the elder people in Bahir Dar city, Northwest Ethiopia 2021.

Variables	Category	Frequency	Percentage (%)
Age	65-74 75-84 >or=85	492 90 43	78.59 14.37 6.87
Sex	Female Male	360 266	57.51 42.49
Religion	Orthodox Muslim Protestant Catholic	454 132 23 17	72.52 21.09 3.67 2.72
Level of Education	No formal education Elementary (1-8) Secondary (9-12) College and University	247 189 102 88	39.46 30.19 16.29 14.06
Marital Status	Married Single Separated Divorced Widowed	349 4 42 113 118	55.75 0.64 6.71 18.05 18.85
Job	Merchant Farmer Daily Labor homemakers Retired Other*	189 32 29 203 134 36	30.19 5.11 4.63 32.43 21.41 5.75
Income	≤2248 >2248	100 526	15.97 84.03
Living condition	With Family Alone Other**	538 47 41	85.94 7.51 6.55

(n=626).

Others*: church, mosque. Others^**^: child, neighbors, and other relatives like sister and brother.

### Clinical and psychosocial factors

Of the study participants, 255 (40.73%) reported having depression. Almost half of the respondents, 307 (49.04%), reported having a poor quality of life, 9 (1.44%) reported having limitations in their daily activities, 71 (11.34%) were at risk of malnutrition, and 183 (29.23%) had at least one chronic disease diagnosed. 292 (46.65%) respondents said they felt lonely, and 44 (7.03%) respondents said they had a weak social network status. For the previous three years, at least one stressful life event was reported by 70 (11.18%) of the participants, and 202 (32.27%) of the participants reported being mistreated. Out of all the participants, 88 (14.06%) had ever used khat, 188 (30.03%) had ever consumed alcohol, and 21 (3.35%) had ever used tobacco (**Table 2**).

**Table 2:** Clinical and psychosocial factors of elderly respondents in Bahir Dar city, Northwest Ethiopia, 2021.

Clinical factors	Category	Frequency	Percentage (%)
Depression	Yes No	255 371	40.73 59.27
Quality of life	Yes No	307 319	49.04 50.96
Chronic disease	Yes No	183 443	29.23 70.77
Functional disability	Dependent Independent	9 617	1.44 98.56
Nutritional status	Malnutrition Risk malnutrition Normal	13 71 542	2.08 11.34 86.58
Social Network	Risk to social isolation No risk of social isolation	44 582	7.03 92.97
Perceive loneliness	Yes No	292 334	46.65 53.34
Geriatrics mistreatment	Yes No	202 424	32.27 67.73
Stressful life event	Yes No	70 556	11.18 88.82
Ever substance use	Khat Tobacco Alcohol	88 21 188	14.06 3.35 30.03
Current substance use	Khat Tobacco Alcohol	66 22 97	10.54 3.51 15.49

(n=626).

### Prevalence of suicidal behaviors

**Figure 2:**
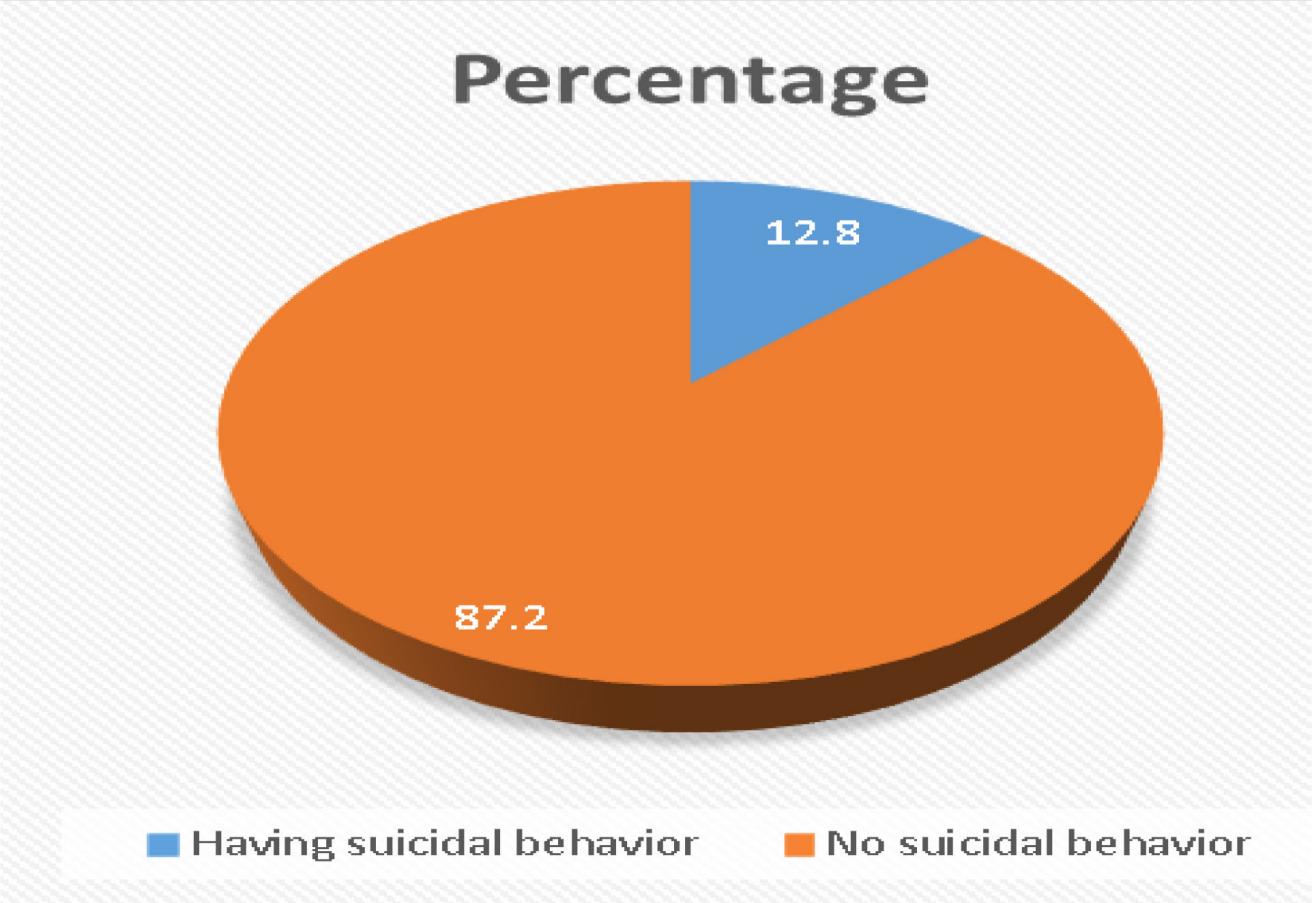
The overall prevalence of suicidal behavior among elders in Bahir Dar city, Northwest Ethiopia, 2021.

The overall prevalence of suicidal behaviors was 12.8% (95% CI; 10.2%, 15.3%) (**Figure 2**). The lifetime prevalence of suicidal ideation, plan, and attempts were 13.90% (95% CI; 8.8%, 16.41%), 8.15% (95% CI; 6.4%, 12.5%), and 1.0%, respectively. The prevalence of suicidal ideation in the past one year was 68 (10.86%); out of those, 54 (8.63%) had once and 14 (2.24%) had twice suicidal ideation at two different points in time. Eighty-nine (14.22%) respondents had the threat of a suicide attempt or they told other people they were going to commit suicide; out of those, 73 (11.7%) once and 14 (2.24%) more than once told others. The likelihood of suicidal behavior in the future was reported by 186 (29.71%) participants (**Table 3**).

**Table 3:** The prevalence of suicidal behaviors among elders in Bahir Dar city, Northwest Ethiopia, 2021.

Variables	Category	Frequency	Percentage (%)
Life time suicidal ideation, intent and/or attempts	Ideation Plan Attempt	87 51 6	13.90 8.15 1.00
Frequency of suicidal ideation in the past one year	Once Twice One year all suicidal ideation	54 14 68	8.63 2.24 10.86
Suicidal threats	Once Twice or more 3-4 times	73 14 2	11.66 2.24 0.32
Likely hood of suicide in the future	No chance at all Rather unlikely Unlikely Likely Rather likely Very likely	97 41 10 32 4 2	15.49 6.55 1.60 5.11 0.64 0.32

(n=626).

### Factors associated with suicidal behavior

The multivariable analysis revealed that suicidal behavior was significantly correlated with several factors, including not being married (AOR: 2.19, 95% CI; 1.18, 4.06), having a risky social network (AOR: 2.25, 95% CI; 1.01, 5.01), having a chronic illness (AOR: 3.03, 95% CI; 1.69, 5.44), having depression (AOR: 4.01, 95%CI; 1.97, 8.17), and experiencing geriatric mistreatment (AOR: 7.81, 95% CI; 4.06, 15.05) (**Table 4**).

**Table 4:** Bivariate and multivariable independent factors of suicidal behavior among elders in Bahir Dar city, northwest Ethiopia, 2021.

Variable	Category	Suicidal behavior		COR(95% CI)	AOR(95%CI)
		No	Yes		
Marital Status	Married Unmarried*	327 219	22 58	1 3.94(2.34-6.62)	1 2.19(1.18-4.06)*
Income	Low High	75 471	25 55	2.86(1.68-4.86) 1	1.07(0.51-2.23) 1
Social Network	Risk Not Risk	22 524	22 58	9(4.72-17.31) 1	2.25(1.01-5.01)* 1
Perceived Loneliness	No Yes	315 231	19 61	1 4.34(2.55-7.53)	1 1.034(0.50-2.16)
Quality of Life	Poor Good	250 296	57 23	2.93(1.76-4.90) 1	1.57(0.67-2.86) 1
Nutrition	Malnutrition Risk	6 49	7 22	11.23(3.6-34.7) 4.32(2.42-7.72)	3.74(0.85-16.43) 1.38(0.68-3.13)
	Normal	491	51	1	1
Chronic Illness*	No Yes	415 131	28 52	1 5.88(3.57-9.70)	1 3.03(1.69-5.44)**
Depression	No Yes	359 187	12 68	1 10.88(5.74-20.6)	1 4.01(1.97-8.17)**
Geriatrics Mistreatment	No Yes	410 136	14 66	1 14(7.73-26.12)	1 7.81(4.06-15.05)**
Stressful Life Event	No Yes	500 46	56 24	1 4.66(2.65-8.20)	1 1.04(0.69-3.13)

(n=626).

1 = reference group, *p<0.05; **p<0.01, COR, crude odds ratio; AOR, adjusted odds ratio.

Unmarried*: include single, separated, divorced, and widowed participants.

Chronic Illness*: CHF, COPD, Diabetes, Hypertension, and others.

## Discussion

Elderly suicidal behavior has a serious detrimental effect on people as well as society. This covers the social, psychological, and physical effects on the elder population, their families, healthcare systems, and communities. Therefore, this study showed that the overall prevalence of suicidal behavior was 12.8% (95% CI: 10.2-15.3%). The lifetime suicidal ideation, plan, and attempt were 13.90%, 8.15%, and 1.0%, respectively. The result was in line with a meta-analysis study in European countries, which shows a 12% (40), a study in China 14.5% (37), and a study done in the United States 12% (20).

Our results, however, were less than those of earlier research that found 15.7% in Brazil (41) and 43% in Austria (42). The possible discrepancy may be a lack of knowledge and attitudes toward suicidal behavior, in which participants may hide; the Ethiopian community cultural and religious beliefs often stigmatize suicide, viewed as morally or religiously unacceptable. Such stigma may discourage individuals from acting on suicidal thoughts or reduce the likelihood of suicide being reported which may contribute to lower reported rates (43, 44). Additionally, those over 60 were included in Brazil’s study (41). With a compression of the one-year prevalence of suicidal ideation at 10.86% and 11%, respectively, the study’s results were comparable to those of the Austrian study (42).

It was also less than the results in Iran (21.07%) (45) and 30.7% in Turkey (46). This may be because the study carried out in Turkey was done in an outpatient psychiatric clinic, and it is commonly known that suicidal behavior is more common in psychiatric patients (47). This is corroborated by a study carried out in the teaching hospital of Jimma University, which found that 28.6% of patients had suicidal behavior (48). The Suicidal Ideation Scale, a tool designed to measure suicidal ideation intensity, individual attitudes toward these thoughts, intention to carry out plans, and factors influencing intention and determination to carry out plans, may also be used as an explanation (49). the information gathered from hand-filled reports from the Mental Health and Suicide Surveillance Systems, which included the death cases in the Iranian study, which may increase the suicide rate (45).

On the other hand, our result was higher than the other studies, including 4.5% in Japan (50) 6% in Hong Kong (28). This might be due to the cultural acceptance of suicide in Ethiopia, whereby the stigma attached to suicide may deter people from reporting it, hence making people less likely to seek help. On the other hand, in European countries, cultural attitudes toward mental health may be more open, thus leading to higher reporting. The other possible difference between the later and current study might be due to the tool used, which was derived from six items of the Geriatric Mental State Examination-Version A and prepared a semi-structured interview designed for elderly subjects; however, the current study used a structured tool (51). Elder care support programs, regular mental health screenings, stigma-reducing education campaigns, and elder abuse reporting systems, which ultimately offer recommendations for integrating mental health into national aging policies and creating legal protections for elders, are some examples of suggested interventions (22). In order to provide a comprehensive framework for preventing elder suicide, multisector collaboration promotes alliances between social service agencies, healthcare providers, and legislators.

Furthermore, this finding was higher than that of a study conducted in Nigeria, which revealed 4.0%, 0.7%, and 0.2%, respectively, of suicidal ideation, plan, and attempts (52). The disparity could be due to the fact that the assessment of suicidal conduct was limited to the period since the respondents turned 65, rather than their entire lives (52). This study took into account the elders’ lifetime of suicidal behavior. Another reason could be that the tool utilized, the Composite International Diagnosis Interview (CIDI), has a low sensitivity score of 0.52.

In this study, the odds of having suicidal behavior were 2.19 times higher in unmarried respondents than in married participants. This may be due to the separated/widowed/divorced individuals who may feel lonely and helpless, thus increase the suicidal tendencies (52, 53). In addition, unmarried individuals have limited social connectedness and poor social networks, which will be associated with suicidal ideation and suicide in later life (7), and social isolation has a strong association with suicidal thoughts and attempts for the elderly (3). The loss of a spouse through death, separation, or divorced has been associated with poorer well-being, loneliness, depression, and suicide (54).

Participants who had poor social networks were 2.25 times more likely to have suicidal behavior than those who had good social networks. This could be due to life stressors and social isolation contributing independently to risk for suicide in later life, whereas social support may help protect against the emergence of suicidal states (55). Establishing and bolstering networks of community support, such as peer support programs and elder care groups, which have been demonstrated to improve mental health and lessen social isolation; encouraging regular mental health screenings and incorporating suicide prevention into primary healthcare, especially in settings where elders are receiving treatment for chronic illnesses or other medical conditions; and reducing stigma by educating families, caregivers, and community leaders about depression, elder abuse, and suicide (56, 57).

Encouraging multispectral collaboration among healthcare providers, social workers, and policymakers to develop a comprehensive suicide prevention framework; promoting elder protection laws that address mistreatment and provide easily accessible reporting mechanisms; and strengthening mental health services for elders by incorporating elder-specific mental health programs into national healthcare strategies. This is supported by a previous study done in Taiwan (17).

Participants with chronic medical illnesses had a 3.03-fold increased risk of suicidal behavior than those who had not. The possible reasons could be due to physical illnesses that are common in late life and may lead to loss of autonomy, isolation, pain, and increased burden on social networks, which will intensify the suicide rates (58). The other reason could be that older persons who experience physical decline and chronic illnesses frequently feel frustrated and powerless, which might raise their risk of suicide. Chronic illnesses like dementia, heart disease, or arthritis can cause people to lose their independence, which can cause mental distress and a feeling of burdensomeness to family members (59). This is consistent with the previous findings in Taiwan (17), and China (60).

The odds of developing suicidal behavior were 4.01 times higher among individuals who had depression when compared to respondents without depression. This might be due to having depressive symptoms reduce the quality of life of older persons and can result in suicidal ideation or behavior (61). The other possible reasons could be suicide behavior in older persons is depression. Suicidal ideas and attempts are more common in older populations with depression, which is frequently underdiagnosed and undertreated. Suicide risk may rise as a result of complex interactions between the biological, psychological, and social components of depression. Depression can be made worse by functional disability, loss of independence, and chronic illness, which can result in pessimism and despair (62). This is consistent with the study done in Korea (63), and China (28).

Those participants who had been mistreated (abuse) by their relatives, family, and friends where there is an expectation of trust were 7.81 times more likely to have suicidal behavior than those had excellently treated (not abused). This could be because their own relatives, family, and friends abused them, as they expected trust from them. Consequently, they may experience sadness, hopelessness, and guilt, and emptiness, which increase the suicidal tendencies (64–66). The finding was supported by a study conducted in on USA (67), and China (68).

### Limitation of the study

The study may not be entirely representative of the Ethiopian population as a whole because it was carried out in urban areas of Northwest Ethiopia. The prevalence and characteristics of suicidal behavior may be influenced by the substantial differences between urban and rural locations with regard to socioeconomic considerations, healthcare availability, and cultural attitudes about suicide.

Recall bias may be explained by the fact that those who do not exhibit suicidal behavior may be less motivated than those who do to recollect past thoughts of suicide events. A cause-and-effect link cannot be shown due to the study’s cross-sectional design. Therefore, longitudinal research is required to investigate causative relationships: causality between depression, elder abuse, and suicide behavior must be established.

Due to the fact that the data was collected through interview-administered, social desirability bias may potentially be an issue. This is because participants may be more likely to give answers that are socially acceptable when answering questions on substance use.

## Conclusion

The extent of suicidal behavior was found to be high in this study. The associated factors of suicide behavior include being unmarried, having a poor social network, having depression, chronic illness, and geriatric mistreatments. Therefore, clinicians should do routine mental health examinations for older persons, focusing on those who have a history of elder abuse or chronic illnesses, as these are major risk factors for suicide behavior. To detect and treat suicidal thoughts in elder populations, healthcare professionals should get culturally appropriate training. For legislators: create policies that address elder abuse by instituting community reporting mechanisms and legal protections for elder citizens, and give top priority to developing national healthcare initiatives that include elder-specific mental health and suicide prevention programs.

## Data Availability

The original contributions presented in the study are included in the article/supplementary material. Further inquiries can be directed to the corresponding author.
